# Water is a biomarker of changes in the cellular environment in live animals

**DOI:** 10.1038/s41598-020-66022-9

**Published:** 2020-06-04

**Authors:** Pratibha Siwach, Evgeniya Levy, Leonid Livshits, Yuri Feldman, Daniel Kaganovich

**Affiliations:** 1 Department of Experimental Neurodegeneration, University Medical Center Göttingen, Walweg 33, 37073 Göttingen, Germany; 2 1Base Pharmaceuticals, Boston, MA 02129 USA; 3 Department of Applied Physics, The Hebrew University of Jerusalem, Edmond J Safra campus, 919041 Jerusalem, Israel; 4 Vetsuisse Faculty, Institute of Veterinary Physiology, University of Zurich, Winterthurerstrasse 260, CH-8057 Zurich, Switzerland

**Keywords:** Materials science, Physics

## Abstract

The biological processes that are associated with the physiological fitness state of a cell comprise a diverse set of molecular events. Reactive oxygen species (ROS), mitochondrial dysfunction, telomere shortening, genomic instability, epigenetic changes, protein aggregation, and down-regulation of quality control mechanisms are all hallmarks of cellular decline. Stress-related and decline-related changes can be assayed, but usually through means that are highly disruptive to living cells and tissues. Biomarkers for organismal decline and aging are urgently needed for diagnostic and drug development. Our goal in this study is to provide a proof-of-concept for a non-invasive assay of global molecular events in the cytoplasm of living animals. We show that Microwave Dielectric Spectroscopy (MDS) can be used to determine the hydration state of the intracellular environment in live *C. elegans* worms. MDS spectra were correlative with altered states in the cellular protein folding environment known to be associated with previously described mutations in the *C. elegans* lifespan and stress-response pathways.

## Introduction

**Figure 1:**
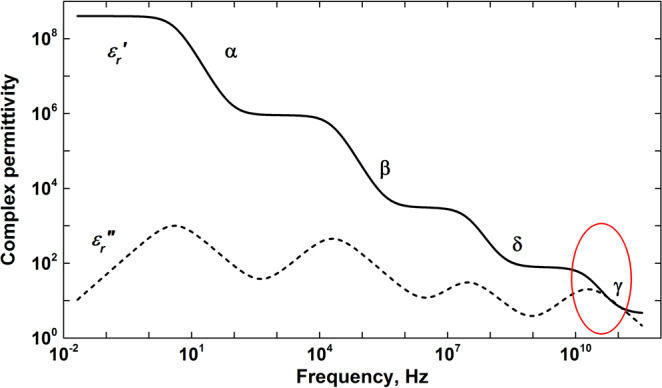
Schematic presentation of the dielectric spectra for biological system (11–13); The dielectric dispersions (α,β, γ,δ) appear in different frequency regions. The α-dispersion, usually found at frequencies below 10 kHz, is not easy to measure accurately because of interference from the electrode polarization (EP) effect. The β-dispersion is due to the interfacial polarization (IP) and is mainly attributed to the existence of the less conducting plasma membrane surrounding the cell. The γ-dispersion usually seen above 1 GHz results from reorientation of water molecules in the cytoplasm and the external medium. In addition, scattered between the β and γ-dispersions there are sometimes small dispersions (called δ-dispersion) that could be attributed to biopolymers and water molecules interacting with biopolymers and membrane surfaces.

The hydrogen bonds formed by water molecules are an essential component to most intracellular molecular events. While it has been traditionally thought that water molecules in the cell are everywhere in excess and that hydrogen bonds form randomly, today there is a greater appreciation of the scarcity of water molecules in many cellular sub-compartments^1–4^. Therefore, there is a pressing need to develop approaches that apply methodology for detecting the hydration states of water to the study of molecular organization of water molecules in live cells. In recent years the molecular structure of the interface between biomolecules and water has been increasingly seen as an important component of sub cellular organization and function^5–7^. Water molecules localized to a hydration shell in the vicinity of a biomolecular “surface” are sometimes referred to as “bound water,” “interfacial water,” or “hydrated water.” These water molecules exhibit dynamic properties that deviate strikingly from those of bulk water^8^. The different states of hydration water span an extremely wide range of time scales for interfacial rearrangements^9^. There are few experimental methodologies capable of assessing this range of time scales concurrently. One of the most versatile is Broadband Dielectric Spectroscopy (BDS)^10^. BDS covers a wide frequency range, from ~10^−6^ Hz to ~10^12^ Hz, enabling tractable exploration of the full spectrum of dielectric relaxation phenomena. It is particularly useful for studying a polar environment like water and water-solvated samples. In biological samples, the dielectric relaxation of water is called γ-dispersion, and falls in the microwave range (between 0.5 and 50 GHz)^11–13^ (Fig. 1).

In this frequency band, the permittivity changes are mainly associated with the response of bulk (free) water. In our recent studies using Microwave Dielectric Spectroscopy (MDS), we discovered that the dynamic balance between bound and free water changes over the course of chronological cellular decline in human red blood cells (RBCs)^14^. Despite the broad implications of these findings, there have been very few studies applying this approach to investigating biologically tractable model organisms. The goal of this paper is to take the first step towards using non-invasive MDS to establish the balance between the bound and unbound intracellular water fractions as a reporter for global stress-associated molecular events.

In order to examine the state of water organization in living organisms, we applied MDS measurements to *Caenorhabditis elegans (C. elegans)* nematodes with distinct genetically determined lifespans and stress-tolerance states^15^. *C. elegans* provide a tremendous advantage for a proof-of-concept study because it is one of the most heavily researched and tractable model organisms, particularly with respect to changes in the cellular environment. The lifespan of *C. elegans* is very short (about three weeks) and stress-associated changes have been well characterized in worms, and have been shown to parallel human stress-induced pathology^16–18^. Furthermore, single point mutations alter the *C. elegans* cellular stress-tolerance parameters in both directions, in easily quantifiable ways^19,20^. Thus, by using worms one can easily compare animals with genetically distinct surrogate markers of different types of stress. The main question that we posed was whether we would be able to use MDS to investigate intracellular hydration states in the cytoplasm of a live organism, particularly an organism in which water organization states can ultimately be used as a correlate for previously-characterized global differences in their levels of cellular stress. Our data suggest that that dielectric relaxation of water can indeed be used as a biomarker for intracellular states associated with stress in *C. elegans*.

## Experimental Details

### Culture and Synchronization of *C. elegans*

All worm strains (N2, daf2(e1370), daf16(mgDf50), hsf1(sy441)) were grown on 10 cm seeded NGM plates. 5 to 7 NGM plates with adult worms (gravid hermaphrodites) were washed with freshly prepared 1X M9 buffer and bleached according to standard protocols^21,22^. Washing and growing embryos: Centrifugation (1690g for 2 min) was performed to sediment worm embryos. Tubes were kept on gentle rotation for 16–18 h at room temperature resulting in a synchronized L1 population after 16 hours. Throughout this study, all the worm strains were maintained and cultured on standard NGM media with OP50 bacteria at recommended temperatures (WT(N2), *daf-16* and *hsf-1* @21 °C *daf-2* @16 °C).

### Sample preparation for Dielectric spectroscopy

L1: worms were centrifuged at 2000g for 2 min. Then supernatant was removed, followed by two washes with fresh filtered M9 buffer. 2 ml of 70% Sucrose was added to 1 ml M9 worm suspension, tapped gently to get homogeneous solution for dielectric measurement. Worms were counted for every sample analyzed.

L4: worms were washed with M9 buffer 3–5 times and were transferred on new seeded NGM plates (15–20 plates per strain were used to avoid over-crowding and food stress). L4 stage was confirmed by stereoscope visualization after 48–72hrs depending on strain. Worms were washed and re-suspended homogenously in 70% sucrose solution before taking dielectric measurement as described earlier^23,24^.

Dauer: To attain L3d stage, L1 worms were retrieved after their dielectric measurements, washed 3–5 times with filtered and fresh M9 buffer and transferred on new non-seeded NGM plates (around 7 plates per strain to facilitate over-crowding and food deficiency for L3d induction) at respective temperatures. After 72 hours when L3d stage was confirmed under stereoscope, worms were washed twice in M9 buffer and suspended in 70% sucrose solution followed by dielectric measurement (as described above).

### Dielectric spectroscopy

Dielectric measurements were carried out in the frequency range from 500 MHz to 20 GHz using a Microwave Network Analyzer (Keysight N5230A PNA-L), together with a Flexible Cable and Slim-Form Probe (Keysight N1501A Dielectric Probe Kit). The calibration of the system was performed with the aid of three references: air, a Keysight standard short circuit and pure water at 25 °C. A special stand for the Slim-Form Probe was designed and combined with a sample cell holder for liquids (total volume ~7.8 mL). The holder was enveloped by a thermal jacket and attached to a Julabo CF 41 oil-based heat circulatory system. The cell was held at 25 °C by the circulator-thermostat with temperature fluctuations less than 0.1 °C. The whole measuring system was placed in an air-conditioned room maintained at 25 ± 1 °C. Spectra at different pH, temperatures, and NaCl concentrations are showed in Supplementary Figs. 1–3 as controls. The MDS measurement for each worm strain was performed at least six times at the indicated time-point. The real and imaginary parts ε′(ω) and ε″(ω) were evaluated using the Keysight N1500A Materials Measurement Software with an accuracy of $\Delta \varepsilon {\prime} /\varepsilon {\prime} =0.05,\,\Delta \varepsilon {\prime\prime} /\varepsilon {\prime\prime} =0.05$^25^.

In order to generate a homogeneous suspension around the Slim-Form Probe we chose to measure worms in high concentrated sucrose solution because it helps to prolong sedimentation time for worms in the suspension (~100 per measurement). High sucrose concentrations do not harm the *C. elegans* and is a standard procedure for washing off bacterial culture^26^.

## Results and Discussion

### Estimating the contribution of water inside worms

In order to measure the gamma dispersion dynamics in *C. elegans*, we were initially faced with the problem of extracting the contribution of the worm cytoplasm to the observed dielectric spectra of the inhomogeneous suspension of nematodes in sucrose buffer. In previous work with Red Blood Cells (RBC) suspensions, the cell size and volume fraction of the RBCs were well known and easy to control^14,27^. The sizes of the *C. elegans* worms, however, are comparable with the size of the open coaxial probe and the field configuration in the sample holder did not allow us to average the signal through the entire suspension. This was further complicated by the variability in individual worm volume fractions (i.e. the precise number of worms per sample). Fortunately, numerous repetitions of dielectric measurements showed that the fitting results are not dependent on the accuracy of the volume fraction estimation. This suggests that in the current study, due to the comparable size of the worms and our Slim-Form Probe, each measurement encompassed one or two organisms. This meant that we were not able to use the conventional approach used for mixture formulas in order to evaluate spectra for one worm. Even knowing the precise number of cells in a single organism, we are not able to estimate the volume fraction of a single cell. However, assuming that in the microwave frequency range there is no contribution of biological components other than water we can posit that its properties are a function of the water inside the worms.

### Dielectric measurements

The experimental complex permittivity spectra of the bulk water can be described by the simple Debye function over a wide temperature range and at frequencies up to 40 GHz^28,29^:

1 $${\varepsilon }^{\ast }(f)=\varepsilon {\prime} (\omega )-i\varepsilon {\prime\prime} (\omega )={\varepsilon }_{h}+\frac{\Delta \varepsilon }{1+i\omega \tau }$$

**Figure 2:**
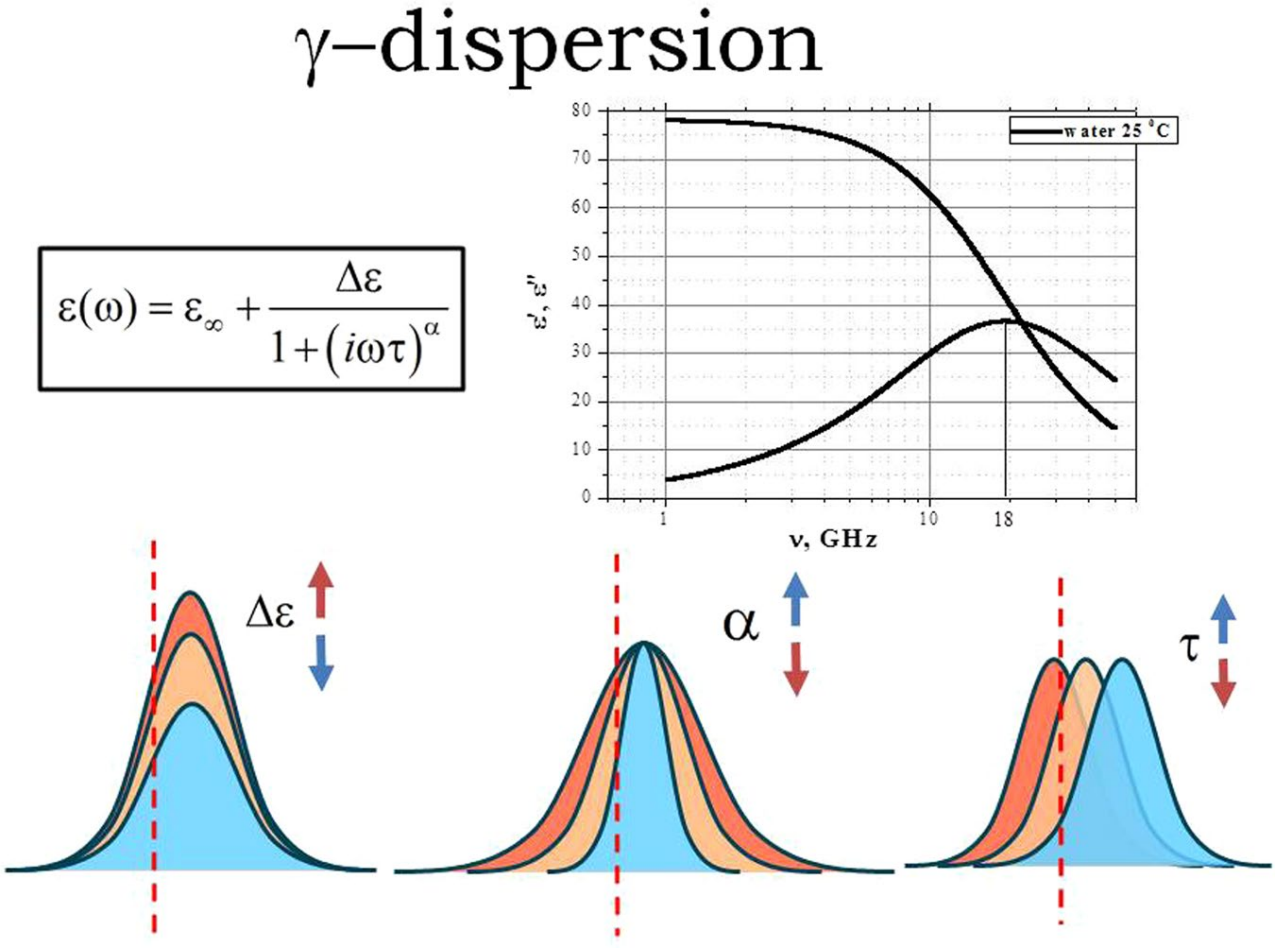
Schematic presentation of CC parameters Δε, τ and α behavior with variation of the balance between bulk and bound water in any aqueous solution or suspension. An increase in Δε indicates that water is released from its bound state and moves to the bulk state. An increase in τ (red shift) suggests that the type of interaction between solvent and solute has a dipole-dipole character, whereas a decrease in τ (blue shift) means that the solute/solvent interaction has an ion-dipole character. A decrease in α indicates that the broadening is wider and the rate of exchange between the hydrated shell and bulk water is higher. An increase in α suggests that the rate of interaction with the solute is decreased.

However, whenever water interacts with another dipolar or charged entity, a symmetrical broadening of its dispersion peak and a change in the attendant relaxation time is induced (Fig. 2)^23,24,30–32^. The origin of the main peak broadening is determined by the dynamics of H-bond network rearrangements in the direct vicinity of solute molecules. The nature of the hydration shell-solute interaction (e.g. dipole-dipole or charge-dipole), can be ascertained by studying the solute concentration dependency of the broadening. In aqueous solutions, the dielectric signature of the hydration shell is negligible and cannot (currently) be directly detected in dielectric experiments^33–37^.

The modification of the water relaxation peak can be described by the phenomenological Cole-Cole (CC) function^38^:

2 $${\varepsilon }^{\ast }(f)={\varepsilon }_{h}+\frac{\Delta \varepsilon }{1+{(i\omega \tau )}^{\alpha }},$$

Here *ε*′ and *ε*″ are the real and the imaginary parts of the complex permittivity, *ω* = *2πf* is the cyclic frequency, and *i*^2^ = −1. The parameter ${\varepsilon }_{h}$ denotes the extrapolated high-frequency permittivity and $\Delta \varepsilon ={\varepsilon }_{l}-{\varepsilon }_{h}$, is the relaxation amplitude or dielectric strength (with the low frequency permittivity limit denoted by ${\varepsilon }_{l}$). The exponent *α* (0 < *α ≤* 1) is a measure of the symmetrical broadening. The relationship between the parameters of the main water peak in aqueous solutions is linked to the nature of the solute and can be integrated into a new phenomenological approach^10,30^ to describe the dynamics of the water/solute interaction (Fig. 2).

The broadening parameter, *α*, is a time fractal dimension, which is controlled by macroscopic physical quantities and reflects the rate of interactions of the diplole relaxation units with their surroundings^39,40^.

### Measurements in live animals

In order to evaluate the potential for dielectric spectroscopy as biomarker for cytoplasmic hydration states, we used the wild-type (WT) Bristol strain (N2) *C. elegans* worms, along with three mutant lines (*daf-2, daf-16* and *hsf-1*) carrying mutations in the insulin/insulin growth factor-like (IIS) signaling pathway, which have a range of genetically programmed life spans and cellular stress-tolerance levels. The DAF-2 gene encodes the IIS receptor, whereas DAF-16 and HSF-1 are downstream transcription factor targets of the kinase cascade regulated by DAF-2. Worms carrying the daf-2 mutation, which suppresses the activity of the IIS receptor, have a significantly extended lifespan and stress tolerance as compared to WT^15^. Additionally, we used two lines in which transcription factors downstream of DAF-2 were knocked down (*hsf-1* and *daf-16*). These transcription factors drive the expression of stress-response transcriptomes, and their absence reduces WT fitness as well as *daf-2* fitness. *daf-2* worms have longer lifespans than WT worms and they display an overall increased tolerance to stress^15^. Hence, *daf-2* worms are resistant to the onset of protein aggregation, including in models of Alzherimer’s and Huntingon’s Diseases^16–18^. Conversely, *hsf-1* and *daf-16* mutants are more sensitive to protein folding stress^41^. Given the difficulty in isolating large homogeneous populations of worms for every measurement, we decided to study the WT and mutant worms at embryonic stages of development. To understand the correlation between dielectric relaxation time and genetically-programmed cytosolic stress tolerance state we subjected live WT and mutant worms to dielectric relaxation measurements at three different larval (post-hatching) developmental stages (i.e., L1, L4 and dauer).

**Figure 3:**
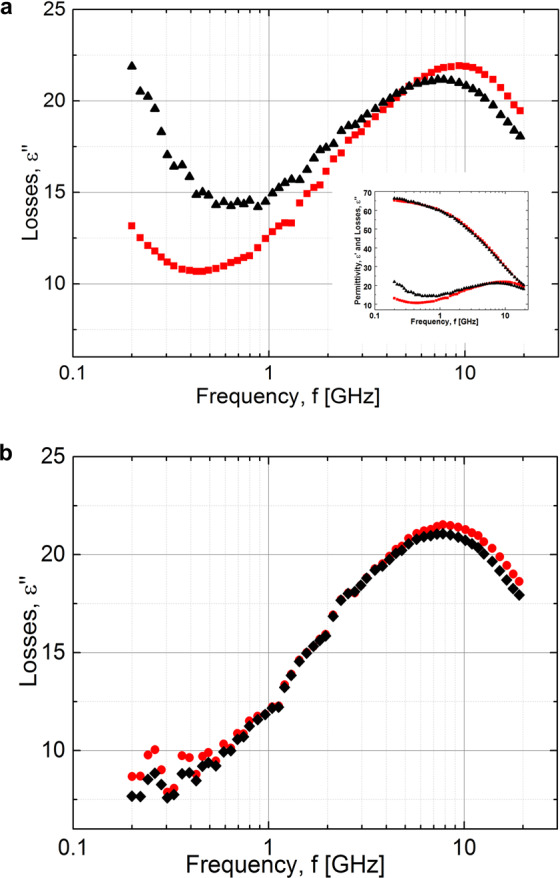
Dielectric losses spectra of C. elegans suspension at 25 °C. (**a**). WT, L1 (black triangles) and dauer (red squares). (In the insert the real and imaginary parts of same spectra). (**b**) WT, L4 (red circles) in comparison to hsf-1 (black rhombi)). The lines represent fitting curves.

The examples of a typical dielectric spectra for worms at 25 °C are shown in Fig. 3. The spectra were fitted using in-house software, Datama^42^, capable of simultaneously modelling both the real and imaginary components of the measured permittivity. The model function used was based on the sum of a CC function (Eq. 1) and a conductivity term −*iσ*/(*ε*_0_*ω*), where *σ* is the electrical *dc* conductivity and ${\varepsilon }_{0}\cong 8.85\,\cdot \,{10}^{-12}\,{\rm{F}}/{\rm{m}}$ is the dielectric permittivity of free space.

### Changes in water hydration state in live worms

**Figure 4:**
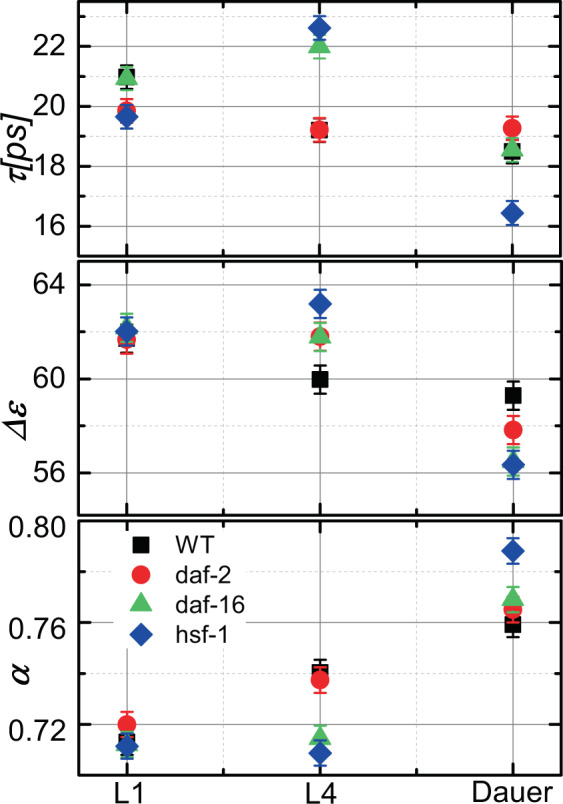
The experimental relaxation times τ, the relaxation amplitude Δε, and the broadening parameter α for *C. elegans* cytoplasm for WT worm and three mutants at different larval stages. Each measurement was performed on 3 separate suspensions of about 100 worms, and each strain (mutants and WT) was measured five separate times. Mean values (±SD) for the total number of measurements are presented as error bars.

Figure 4 shows the relaxation time (*τ)*, the broadening parameter (*α)*, and the dielectric strength (Δ*ε)*, of the WT worm cytosol compared to mutants over the course of larval development. We found it reassuring that at L1 all animals showed very similar Cole-Cole fitting parameters, which then diverged for different mutants in a clear and reproducible manner over the course of their larval development. WT worms show a subtle decrease of water relaxation time corresponding to an increase of dipole ionic interaction (blue shift)^24^. This is coupled with a decrease in dielectric strength that represents an increase of the bound water fraction over time. The broadening parameter α increased, indicating a decrease in the bulk water exchange rate, i.e. prolonged interaction with the hydration shell^43,44^ Together, these parameter trends suggest that during normal worm development, there is a decrease in bulk water and conversely an increase in hydration. This is consistent with previous reports suggesting an increase in protein aggregation in lifespan-restricted mutant worms^45^. Our data would further argue that IIS pathway mutations decrease the amount of free water in the cytoplasm, potentially making it less diffusive^46^. This observation also has implications for the formation of membraneless organelles^47–52^.

Remarkably, *daf-2* worms showed a nearly static relaxation time (*τ)* for their main water peak over the course of the entire larval development. This corresponds to their dramatically (~3 fold) decreased rate of mortality. The for other two parameters, broadening (*α)*, and dielectric strength (Δ*ε), daf-2* worms remained static till L4 with a WT-like trend also in dauer stage. Once again, the behavior of these parameters is highly consistent with the known stress-tolerance state of the *daf-2* worms. *daf-2* worms may have a slightly slower developmental rate than WT worms, since it was grown at a lower temperature; hence it is not clear that they were at the same developmental state as the other worms that were analyzed. Therefore, it is impossible to know whether the effect of the *daf-2* mutation on the relaxation time, broadening, and dielectric strength parameters was due to their developmental state or their IIS-driven cytoplasmic stress tolerance state. In either case, these parameters appear to reflect the fitness state of the cytoplasm in a quantitative way.

What remained was to examine the shorter-lived stress sensitive mutant worms, in order to determine whether they display the opposite trend of long-lived *daf-2* worms. We therefore carried out dielectric measurements in *daf-16* and *hsf-1* knock-down mutant strains, both of which display lower fitness than WT, but have been shown to exhibit different stress-tolerance characteristics^16–18^. As with *daf-2* worms, *daf-16* worms showed dramatically different broadening parameter trends as compared to WT. Initially, *daf-16* worms showed a strong red shift, indicating an increase in dipole-dipole interactions^23,27,32^. Interestingly, over developmental progression the relaxation parameter changes to a blue shift, suggesting that at dauer stage the cellular interior of *daf-16* worms exhibits dramatic ionization resulting in a decrease of water relaxation time. The contribution of the bulk water in that case is decreased (Δε falls in L3d as well). Strikingly, the broadening is nearly static during the first day (L1) and then increases with during development. *hsf-1* mutant worms show a similar broadening pattern, which in their case is even more pronounced. Interestingly, *hsf-1* mutants deviate dramatically from *daf-16* in their relaxation parameter and the peak broadening when the worm became dauer larvae. Together, these data indicate that the relaxation time of intracellular water can be an accurate predictor of the cellular fitness state of a simple organism.

In future experiments, it will be essential to propel this approach further, initially in *C. elegans*, and eventually in mammals. One of the technologically-imposed limitations of the current study is that we were only able to perform measurements in *C. elegans* over the course of the first week of life, whereas their lifespan ranges from ~20 days for WT to ~40 days for *daf-2*. This particularly an issue for the *daf-2* strain, which may be developmentally out of synch with the other strains including WT. The reason that our measurements were limited to the larval development is that very high numbers of identical worms were needed to perform the measurement with the current probe. Additionally, due to current technical constraints the measurements had to be carried out at 25 °C, which can potentially cause a mild heat-shock response in the live *daf-2* worms, which were grown on 16 °C. For future analyses including those of much older worms, we will need to develop a microfluidic microwave probe capable of single-worm measurements at the desired time and temperature. Such an innovation will enable us to bridge between the current proof of concept study and a development of a *bona fide* biomarker for cellular stress.

Here, our goal was to validate an approach that examines the intrinsic hydration state of the cytoplasm, as a potential biomarker for measuring the fitness state of the cytoplasm, which is thought to be a key correlate of organismal fitness. MDS has previously been successful at measuring the hydration state of Red Blood Cells (RBCs), as a function of their chronological decline (time in storage)^27,31,32^. Strikingly, in our current study the MDS approach was also successful at predicting the stress tolerance of *C. elegans* worm mutants, which display highly consistent differences in cytoplasmic state at the same chronological age. Looking to the future, if the Dielectric Spectroscopy approach can be adapted to human samples, it will have the potential to be used as a proxy for intracellular health and will enable further exploration of interventions that can increase the human health-span.

## Conclusions

One of the main challenges in the biomarker field is the application of accurate biophysical tools to the assessment of cellular states in live tissues. Biomarkers are essential for assessing the biological fitness of a patient, predicting vulnerability to disease, and indicating treatment. A biomarker that can be assessed easily and quickly at different points in time is also essential for clinical trials aimed at developing pharmaceutical interventions.

Our goal was to validate an approach that examines the intrinsic molecular state of the cytoplasm, as a potential future biomarker for measuring cellular fitness. Strikingly, our approach was successful at predicting the protein folding stress-tolerance state of *C. elegans* worm mutants, which display highly consistent differences in stress tolerance during early development.

## Supplementary information

Supplementary Dataset 1.
