# Supplementary material for: Water is a biomarker of changes in the cellular environment in live animals
Source: Sci Rep. 2020 Jun 4;10:9095. doi: 10.1038/s41598-020-66022-9 (PMC7272622; doi:10.1038/s41598-020-66022-9)
Supplement: Supplementary file 1 — Supplementary Dataset 1. [file 41598_2020_66022_MOESM1_ESM.docx]

**Water is a biomarker of changes in the cellular environment in live animals**

Pratibha Siwach, Evgeniya Levy, Leonid Livshits , Yuri Feldman and Daniel Kaganovich

**Supplementary materials**

**Figure S1** The dielectric spectra of water in a broad temperature range (4-37 degrees). The spectra follow the classical Debye Model, where α=1^1-4^

**Figure S2** Dielectric spectra of water at different pH at 25^0^C. Measurements were conducted in the frequency range from 500 MHz to 40GHz using a Microwave Network Analyzer (Keysight N5234B PNA-L), together with a Flexible Cable, Slim-Form Probe and ECal module (N4692A Electronic Calibration Module). The measurement procedure was same as described in the paper. The relevant spectra are not sensitive to pH variations in the interval from 5 to 9.

**Figure S3** Dielectric spectra for different concentration of NaCl (aqueous solutions at 25^0^C). The measurements were conducted in the frequency range from 300 MHz to 50GHz using the Microwave Network Analyzer (Agilent N5245A PNA-X), together with a Flexible Cable and Slim-Form Probe. The measurement procedure described in ^5^. Simple organic solvents and vegetable oils usually are non-polar liquids with very small dielectric constants form 1.88 for C_6_H_14_ to 2.29 for C_4_H_8_O_2_ and have dependence on frequency and temperature^6^.

1 U. Kaatze & Y. Feldman. *Meas. Sci. Technol.* **17** (2006).

2 W. J. Ellison. *J. Phys. Chem.Ref. Data* **36** (2007).

3 Popov, I., Ishai, P. B., Khamzin, A. & Feldman, Y. The mechanism of the dielectric relaxation in water. *Physical Chemistry Chemical Physic* **18**, 13941 - 13953 (2016).

4 Kaatze, U. Complex permittivity of water as a function of frequency and temperature. *J Chem Phys Eng Data* **34**, 371-374 (1989).

5 Levy, E., Puzenko, A., Kaatze, U., Ben Ishai, P. & Feldman, Y. Dielectric spectra broadening as the signature of dipole-matrix interaction. II. Water in ionic solutions *J. Chem. Phys.* **136**, 1145031-1145036, doi:10.1063/1.3691183 (2012).

6 Wohlfarth, C. *Static Dielectric Constants of Pure Liquids and Binary Liquid Mixtures*. (Springer-Verlag 2008).
